# Serologic Evidence of Scrub Typhus in the Peruvian Amazon

**DOI:** 10.3201/eid2308.170050

**Published:** 2017-08

**Authors:** Claudine Kocher, Ju Jiang, Amy C. Morrison, Roger Castillo, Mariana Leguia, Steev Loyola, Julia S. Ampuero, Manuel Cespedes, Eric S. Halsey, Daniel G. Bausch, Allen L. Richards

**Affiliations:** Kantonsspital Baden, Baden, Switzerland (C. Kocher);; Epidemiology, Biostatistics, and Epidemiology Institute Travel Clinic, Zurich, Switzerland (C. Kocher);; US Naval Medical Research Unit No. 6, Lima and Iquitos, Peru (C. Kocher, A.C. Morrison, R. Castillo, M. Leguia, S. Loyola, J.S. Ampuero, E.S. Halsey, D.G. Bausch);; US Naval Medical Research Center, Silver Spring, Maryland, USA (J. Jiang, A.L. Richards);; National Institute of Health, Lima (M. Cespedes)

**Keywords:** scrub typhus, serologic analysis, ELISA, immunofluorescence assay, IFA, PCR, Orientia tsutsugamushi, bacteria, rickettsia, vector-borne infections, zoonoses, Peruvian Amazon, Peru

## Abstract

Using a large, passive, febrile surveillance program in Iquitos, Peru, we retrospectively tested human blood specimens for scrub typhus group orientiae by ELISA, immunofluorescence assay, and PCR. Of 1,124 participants, 60 (5.3%) were seropositive, and 1 showed evidence of recent active infection. Our serologic data indicate that scrub typhus is present in the Peruvian Amazon.

Infections with scrub typhus group orientiae (STGO) are a common and widespread cause of fever in Asia, the western Pacific, and northern Australia (the tsutsugamushi triangle). The causative pathogen, *Orientia tsutsugamushi*, is transmitted by the larval stage of trombiculid mites of the genus *Leptobromidium* (chiggers). Clinical manifestations can be mild and unspecific, but complications include jaundice, meningoencephalitis, myocarditis, and interstitial pneumonia leading to acute respiratory distress syndrome and renal failure (*1*).

Epidemiologic studies in Southeast Asia have identified rickettsial illness (especially scrub typhus and murine typhus) as leading causes of treatable undifferentiated febrile illness, although they are often misdiagnosed as malaria, dengue, or typhoid fever (*2*). The causative agent of scrub typhus was not believed to exist outside the tsutsugamushi triangle until *Orientia* spp. were identified by serologic and molecular methods in febrile patients from the Middle East (*3*) and Chile (*4*). Recently, more autochthonous cases of scrub typhus were reported from Chiloé Island in southern Chile, suggesting endemicity in the area (*5*). Therefore, we conducted a study to obtain serologic evidence of scrub typhus in the Peruvian Amazon.

## The Study

This study was conducted in Iquitos, which is located in the Amazon forest in the Department of Loreto in northeastern Peru, where ongoing epidemiologic studies on febrile illness and rickettsial disease (*6**,**7*) have been conducted. STGO testing was nested in an ongoing febrile surveillance study (NMRCD.2010.0010), which was approved by the US Naval Medical Research Unit No. 6 Institutional Review Board (Lima, Peru). The current study protocol was approved by the US Naval Medical Research Unit No. 6 Institutional Review Board in compliance with all applicable federal regulations governing the protection of human subjects.

Febrile surveillance was conducted in 12 health facilities (3 hospitals and 9 outpatient clinics; 2 of the 12 were military facilities) distributed across 4 districts of Iquitos. Febrile patients who fulfilled the inclusion criteria (axillary temperature >37.5°C, duration of illness <5 days, and age >5 years) were asked to participate in the study. An acute-phase blood sample was collected at the time of enrollment, and a convalescent-phase blood sample was obtained 10–30 days later. These samples had already been used for detection of other pathogens (mainly dengue virus and other arboviruses), and results and testing methods have been reported (*8*).

We retrospectively screened convalescent-phase blood samples obtained from participants enrolled during 2013 by using an STGO-specific IgG ELISA. We screened convalescent-phase samples for IgG against a mixture of whole-cell antigen preparations from Karp, Kato, and Gilliam strains of *O. tsutsugamushi* in an STGO-specific IgG ELISA as described (*9**–**11*). For convalescent-phase samples with net absorbance >0.500 at a 1:100 serum dilution, we subsequently assessed STGO IgG ELISA titer (range 1:100–1:6,400) with their paired acute-phase samples side-by-side to determine their endpoint titers. We considered samples with a net total absorbance >1.000 for serum dilutions 1:100, 1:400, 1:1,600, and 1:6,400 titer positive. We classified samples with a seroconversion or a >4-fold increase in IgG titer between acute-phase and convalescent-phase blood samples and minimum titer of 1:400 in the convalescent-phase sample as indicative of active rickettsial infection (*11*).

Samples with evidence of active infection were then confirmed by immunofluorescence assay (IFA) with the *Orientia* MIF IgG Kit (Fuller Laboratories, Fullerton, CA, USA) and Karp, Kato, Gilliam, and Boryong strains of *O. tsutsugamushi* according to the manufacturer’s instructions. We further tested acute-phase samples with evidence for active infection by PCR to identify the causative pathogen.

We extracted DNA from whole blood samples by using QIAmp DNA Mini Kits (QIAGEN, Valencia, CA, USA) following the manufacturer’s instructions except that a final elution volume of 100 μL was used. Samples were stored at −80°C. We assessed DNA samples from persons with evidence for recent active disease by using a quantitative PCR specific for the *O. tsutsugamuchi* 47-kD antigen gene as reported (*12*).

During 2013, we enrolled 1,497 participants in the main study. Of these participants, 1,124 had paired serum samples. Results of the STGO-specific IgG were positive for 60 (5.3%) of 1,124 convalescent-phase serum samples with a titer >1:400. One participant had a >4-fold increase in titer (acute-phase sample titer 1:400, convalescent-phase sample titer >6,400) and was confirmed by IFA as showing a >4-fold increase in titer for 3 of 4 strains of *O. tsutsugamushi* (Karp, Gilliam, and Boryong) and a 2-fold increase in titer for the Kato strain. A test result of the acute-phase sample for *Orientia* sp. DNA was negative. This case-patient was a 22-year-old soldier stationed in a rural military camp at the time of illness. He had fever, chills, malaise, muscle pain, nausea, and vomiting but no rash or jaundice. The patient had not traveled in the 2 weeks before illness and therefore could not have contracted the infection elsewhere.

## Conclusions

We performed a retrospective systematic analysis for the presence of scrub typhus in Iquitos, Peru, by testing blood samples collected prospectively during unspecific acute febrile illness. Although definitive molecular evidence of *Orientia* spp. infection was not found, our serologic evidence strongly suggests the presence of this pathogen in tropical areas of Peru.

Seroprevalence among febrile illness patients was low, especially because most patients tested came from urban rather than rural areas. In addition, our study design could further underestimate scrub typhus because we only included patients with fever for <5 days, whereas the mean time to signs of scrub typhus has been reported as 8.2 days (*13*). Although the 1 patient with ELISA and IFA evidence of seroconversion showed a negative result by PCR, a negative PCR result during the acute phase of scrub typhus is not uncommon, even when testing is performed on whole blood or buffy coat samples (*14*). In addition, whether the causative agents of scrub typhus in South America are recognized by the primer set used in the quantitative PCR is unknown (*4*).

Cross-reactivity with other *Rickettsia* spp. does not seem to be a concern. The *Orientia* spp. ELISA using the immunodominant genus-specific, 56-kDa, type-specific antigen (outer membrane protein [Omp]) does not react with antibodies produced during typhus group rickettsiae (TGR) and spotted fever group rickettsiae (SFGR) infections because rickettsiae lack the 56-kDa type-specific antigen. Similarly, antibodies produced against *Orientia* spp. infection do not react against SFGR and TGR ELISA antigens (lipopolysaccharides OmpA and OmpB), because *Orientia* spp. do not contain these antigens (*15*). As expected, screening results for SFGR and TGR (*7*) were negative for the case-patient we report.

Dengue virus and other arboviruses were excluded (by PCR or virus isolation) as alternative causes of fever. Furthermore, study participants had probably already received over-the-counter medications, including antimicrobial drugs, which would account for false-negative PCR results. According to the prospectively collected clinical data, a typical rash was not documented for the case-patient we report. However, because this surveillance study was not directed toward scrub typhus features, a small eschar could have been easily missed.

This study raises many new issues, such as possible local reservoirs and vectors. The presence of trombiculide mites in Peru has been reported in other areas (*16*), but this species has not been studied as a local vector for pathogens. The case-patient we report was in the military and stationed in a rural camp. Therefore, he was regularly exposed to typical vegetation that supports the presence of mites. Our results and those from studies in Chile (*4**,**5*) indicate a need for a more expansive survey for evidence of scrub typhus, not only in Peru, but throughout South America.
